# Part 1 – Coronary angiography with gadofosveset trisodium: a prospective feasibility study evaluating injection techniques for steady-state imaging

**DOI:** 10.1186/s12872-015-0176-0

**Published:** 2015-12-22

**Authors:** Mark A. Ahlman, Fabio S. Raman, Scott R. Penzak, Jianing Pang, Zhaoyang Fan, Songtao Liu, Neville Gai, Debiao Li, David A. Bluemke

**Affiliations:** Radiology and Imaging Sciences - National Institutes of Health Clinical Center, Bethesda, MD USA; Department of Pharmacotherapy, University of North Texas, Fort Worth, TX USA; Bioengineering, Cedars-Sinai Medical Center, Los Angeles, CA USA

**Keywords:** Gadofosveset trisodium, MS-325, Gadolinium-based intravascular contrast agent, Whole-heart coronary magnetic resonance angiography, Navigator-based angiography, 3.0 Tesla, Image quality, Respiratory motion correction

## Abstract

**Background:**

The purpose of this study was to define an optimal injection protocol for 5–10 min duration navigator-based coronary MR angiography using an intravascular gadolinium-based contrast agent (GBCA), which is better suited for steady-state coronary MR angiography than conventional GBCAs.

**Methods:**

Using projections from pharmacokinetic models of the intravascular concentration of gadofosveset, a dual-injection protocol was formulated and tested on 14 healthy human subjects. Modified Look-Locker inversion recovery (MOLLI) sequences were used for T1 mapping at 3 Tesla to evaluate the concentration of tracer in the aorta over the scanning interval.

**Results:**

Pharmacokinetic models for a bolus plus slow infusion technique at a 5, 10, and 15 min steady state intravascular concentration was compared to single bolus curves. The 70 %/30 % bolus/slow infusion technique resulted in the highest intravascular concentration over a 5 min scan duration. Similarly, the 60 %/40 % bolus/slow infusion technique was projected to be ideal for image acquisition duration of 5–10 min. These models were confirmed with T1 maps on normal volunteers. Arterial-venous mixing of contrast was achieved within 90 s of the beginning of the bolus.

**Conclusions:**

Gadofosveset injection is optimized for the lowest intravascular T1 time for 5–10 min duration MR angiography by bolus injection of 60–70 % of the total dose followed by slow infusion of the remainder of the total dose. This protocol achieves rapid and prolonged steady state intravascular concentrations of the GBCA that may be useful for prolonged image acquisition, such as required for navigator-based coronary MR angiography at 3 Tesla.

**Trial registration:**

ClinicalTrials.gov identifier: NCT01130545NCT01130545, registered as of May 25, 2010.

## Background

Although 3.0 Tesla (3T) magnetic resonance angiography (MRA) can be achieved during a breath-hold with administration of a GBCA, very high-resolution images of coronary or other vessels are not readily accomplished within a breath-hold. Thus, navigator based coronary MRA has been developed [1]. Acquisition time with navigator based MRA is can be high (>10 min), depending on the efficiency of the navigator sequence (typically 30–50 %).

For coronary artery imaging at 3T, the balanced steady-state free precession (bSSFP) technique is less robust and the overall contrast to noise ratio advantage over 1.5 Tesla is diminished [2]. As an alternative, the success of contrast enhanced coronary angiography at 3T had been demonstrated [3] and the addition of 3D non-Cartesian acquisition with 100 % imaging efficiency can reduce imaging time to less than 10 min [4–6]. However even this shorter acquisition time is much longer than the intravascular residence time of common GBCAs [7]. Following bolus injection, extracellular distribution of common GBCAs results in rapid loss of vascular signal and diminished contrast to noise ratio due to enhancement of surrounding structures [7, 8]. Compared to other GBCAs, Gadofosveset (Ablavar®, Lantheus Medical Imaging, North Billerica, MA, USA) has a high intravascular residence time due to albumin binding compared to other GBCAs [9], which is theoretically well suited for this application. Gadofosveset and gadobenate (MultiHance®, Bracco Diagnostic Inc., Singen, Germany) have previously been compared for myocardial enhancement using a slow infusion technique [10], and coronary MRA with gadofosveset has been studied using navigator-based approaches using a single bolus injection [8]. Owing to work investigating alternate infusion techniques with GBCAs with low intravascular residence time [11], the same techniques have not been investigated with gadofosveset, which may be further optimized by a dual injection technique rather than with a single bolus infusion.

The purpose of this study was to determine the parameters for a dual injection protocol for gadofosveset to achieve a steady intravascular T1 signal in vivo in order to optimize imaging for MR acquisition times under 10–15 min, such as with whole-heart coronary MRA at 3T [8, 12–14]. In this study, we performed pharmacokinetic simulations of gadofosveset followed by in vivo studies to measure the T1 values of the arterial vascular compartment.

## Methods

### Study population

This study was conducted at the Clinical Center at the National Institutes of Health (Bethesda, Maryland, United States). The National Heart, Lung, and Blood Institute review board approved the study and informed consent was obtained from all study participants (ClinicalTrials.gov Identifier: NCT01130545). Fourteen eligible subjects were <50 years of age, and were without cardiovascular, renal, or liver disease. Standard exclusion criteria included metallic implants, claustrophobia, impaired glomerular filtration, or any condition or situation that precluded the safety of MR scanning. Weight, height, and heart rate were recorded for all subjects at the time of imaging, as well as serum creatinine and blood pressure. Glomerular filtration rate and body mass index were calculated. Subjects were recruited within a 6-month time period and were permitted to have initial or repeat scanning in the absence of MR contrast administration within the last 30 days.

### Pharmacokinetic modeling

Gadofosveset plasma concentrations vs. time profiles were simulated using WinNonlin pharmacokinetic software, version 5.0 (Pharsight Corporation, Mountain View, CA, USA). Using a 100 % bolus (no slow infusion phase) as a reference, simulated pharmacokinetic data were systematically assessed for their ability to maintain relatively constant gadofosveset plasma concentrations for a 5, 10, and 15 min durations with a bolus followed by a slow infusion phase. Simulations were conducted using initial pharmacokinetic parameter estimates that were based upon the biphasic nature (two compartment open model) of contrast disposition reported in the manufacturer’s prescribing information for gadofosveset. The simulation parameters sought to achieve a steady state T1 of the vascular compartment of less than 200 msec for at least 5 min, governed by the following equation:

1 $$ \frac{1}{\mathrm{T}{1}_{\mathrm{post}}}=\frac{1}{\mathrm{T}{1}_{\mathrm{pre}}}+{\mathrm{r}}_1\cdot \left[{\mathrm{C}}_{\mathrm{b}}\right] $$

In equation (1), pre-contrast T1 (T1_pre_) at 3 T in the blood was assumed to be 1664 ms [15]. The relaxivity (r_1_) of gadofosveset is 9.9 (mmol.s)^−1^ at 3 T in blood plasma [16]. [C_b_] refers to the contrast concentration in blood. [C_b_] = [C_p_] × (1-[Hct]) where [Hct] refers to the hematocrit which was assumed to be 40 % while [C_p_] is contrast concentration in plasma. Using equation 1, post-contrast blood T1 values were calculated for reference to the experimentally determined plots and to the in vivo behavior of the agent.

The gadofosveset vial injection concentration was 0.25 mmol/mL. Dilution of the agent with normal saline to a final volume of 50 mL was intended to standardize and simplify the preparation of the injection protocol. This volume was used for both bolus and slow injection phases; therefore, the final concentration was variable according to the prescribed weight-based dose. The bolus phase of administration was set to a maximum of 1.5 mL/s. The lower limit of the slow infusion rate was set by the MRI power injector and was 0.04 mL/s.

The time required for arterial-venous mixing coupled with the time required for bolus contrast injection could not be accurately modeled by pharmacokinetic software because of the expected inter-subject physiologic variation. Therefore, the assumption of immediate injection and equilibrium were required to limit computation to the two-compartment model, requiring further evaluation of the in vivo behavior at these phases.

### In vivo evaluation

We sought to optimize injection parameters for MRA duration of 5–10 min In vivo. Human testing was performed using parameters derived from the pharmacokinetic simulations (70 %/30 % *n* = 14 and 60 %/40 % *n* = 5), and was compared to the in vivo behavior of a baseline single (100 %, *n* = 3) bolus protocol. Injection protocols were programmed on a Spectris Solaris EP (MEDRAD Inc., Pittsburgh, Pennsylvania, USA) MR compatible power injector. Following standard localizer sequences and injection of gadofosveset, a 4-chamber, single-slice, 11-heart beat modified Look-Locker inversion recovery (MOLLI) sequence was acquired at 45–60 s intervals during a breath hold on a 3T Verio (Siemens Medical Solutions, Erlangen, Germany) using body matrix coils [17]. MOLLI parameters included TR/TE 2.4/1.03 ms; flip angle 35°; TI >125 ms with 80 ms increment; 1002 Hz bandwidth per pixel. Eight images of differing inversion times were acquired with these parameters for a single slice using generalized auto-calibrating partially parallel acquisitions (GRAPPA) factor of 2. Prospective ECG triggering was used for imaging the heart at end diastole.

### Image processing and analysis

**Fig. 1:**
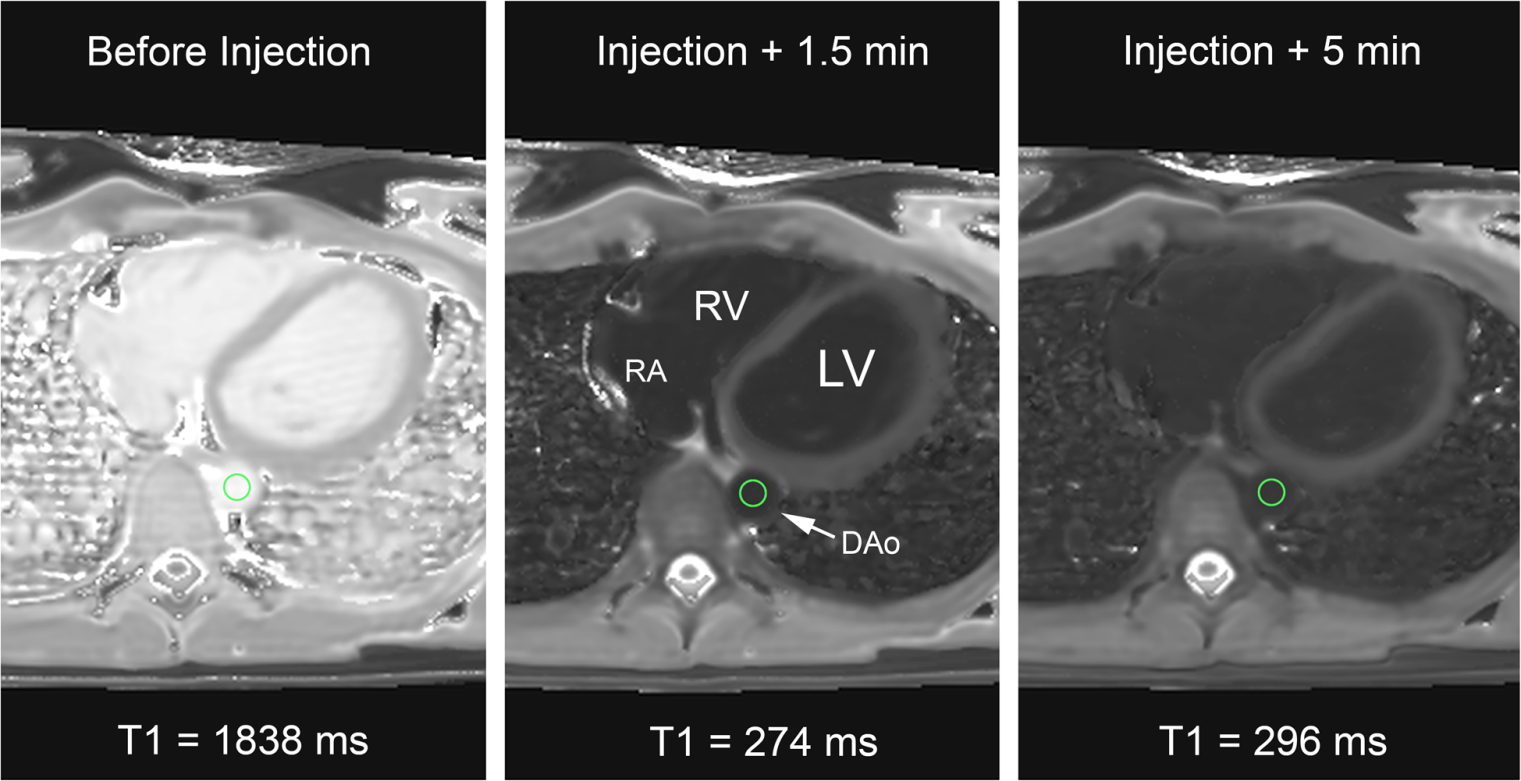
Example of axial MOLLI images acquired of the heart. T1 time (in milliseconds) were derived from T1 maps using the MOLLI pulse sequence in the descending aorta (DAo, white arrow) before injection and at subsequent time points after injection. *MOLLI* – modified Look-Locker inversion recovery, *LV* left ventricle, *RV* right ventricle, *RA* right atrium, *DAo* descending aorta

The gadolinium concentration of the aorta was taken to be similar to the coronary arteries but more accurately determined due to larger size, and did not carry the risk of measurement of trabeculations, as would be the case for ventricular lumen measurement. MOLLI sequences were used to form T1 maps to measure absolute T1 times in the descending aorta using the program QMass MR ver 7.2 (Medis, Raleigh, North Carolina, USA) using regions of interest shown in Fig. 1. Plots of time versus T1 values were used to determine the time to achieve steady state T1 time, and a consensus of 2 reviewers was used to record the time of equilibrium for each scanning instance. Percent change in T1 time from the point of early equilibrium and 5 and 15 min thereafter was recorded, with the intent to maintain T1 time deviation to less than 10 % over intended scan durations. Percent deviation in T1 time and following equilibrium is expressed as the mean with the min-max range, and other metrics are reported as the mean ± the standard deviation. Mann-Whitney testing was used to measure difference in median T1 time over a given scanning interval between predicted and in vivo measurements following equilibrium, with a *p* <0.05 for significance.

## Results

### Pharmacokinetic modeling

**Fig. 2:**
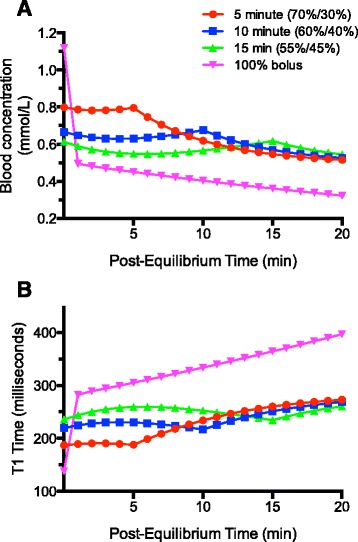
Pharmacokinetic model for the intravascular concentration of gadofosveset. Using various dual injection techniques, a fixed percentage of the total volume is injected as the bolus phase, followed by remainder as a slow infusion over a targeted duration to maintain a steady state concentration. A 100 % bolus is also plotted for reference. The 2-compartment model assumes immediate injection and arterial-venous mixing (equilibrium)

Shown in Fig. 2, the baseline model was 100 % bolus infusion at 1.5 mL/s for gadofosveset dose of 0.12 mL/kg or 0.03 mmol/kg (8.4 mL for a 70 kg subject, approximately 11 s infusion). Using the 100 % bolus approach, blood concentration of gadofosveset was predicted to drop rapidly to 40 % (Fig. 2, 1 min after equilibrium) of maximum after the initial peak vascular concentration. This was followed by a plateau phase beginning about 1 min after the beginning of the infusion (Fig. 2, “100 % bolus” curve), where T1 time increased to more than than 10 % of its baseline equilibrium value for a theoretical 5-minute scan duration.

Administration of 70 % of the total gadofosveset dose as a bolus (1.5 mL/s) followed by 30 % of dose administered as a slow infusion (0.04 mL/s) showed approximately 5 min duration of a relatively high intravascular gadofosveset concentration (approximately 70 % of the 100 % bolus curve) (Fig. 2, 5 min - 70 %/30 % protocol).

Targeted to achieve a constant concentration of gadofosveset over 10 min, a 60 %/40 % (bolus/slow infusion) dosing split showed low variation in intravascular gadofosveset concentration; however, predicted overall concentrations were lower with this approach (0.63–0.68 mmol/L).

For a 15-minute scan duration, a 55 %/45 % (bolus/infusion) dosing split predicted the least variability in intravascular gadofosveset concentration. Overall concentrations were lower with this approach (0.53–0.56 mmol/L), which were insufficient compared to 70 %/30 % and 60 %/40 % protocols, and were therefore not tested in vivo.

### In vivo evaluation

Subject characteristics are shown in Table 1. Gadofosveset dosing was calculated using the subject’s body weight at time of scanning. Twenty-two scanning instances were accomplished with 14 volunteers.

**Table 1:** Subject demographics

Demographics	
Age (years)	34.0 ± 7.1
Male n (%)	5 (36 %)
Height (m)	1.7 ± 0.1
Weight (kg)	71.9 ± 14.1
Body mass index (kg/m^2^)	23.6 ± 2.6
Hematocrit (%)	44.0 ± 3.3
Creatine (mg/dL)	0.88 ± 0.17
GFR^a^ (mL/min/1.73 m^2^)	104.0 ± 15.3
Heart rate (bpm)	65.4 ± 3.9
Systolic blood pressure (mmHg)	118.8 ± 6.8
Diastolic blood pressure (mmHg)	69.0 ± 9.0
Contrast administration	
Dose (mL)	9.0 ± 2.1

^a^GFR was calculated using the chronic kidney disease epidemiology collaboration (CKD-EPI) formula. Because subjects could be imaged more than once; age, gender, and height were calculated once, whereas other measurements were calculated per imaging visit

*GFR* glomerular filtration rate

**Fig. 3:**
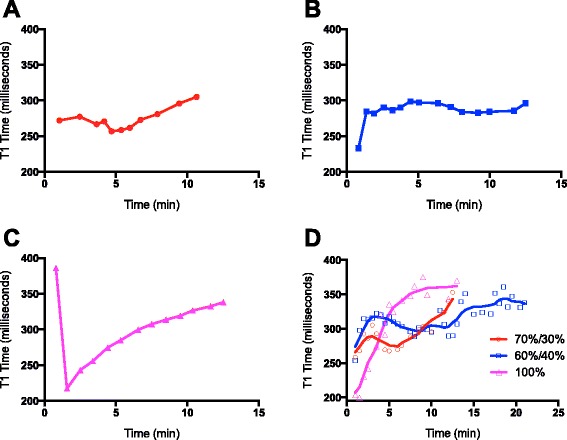
In vivo behavior of dual-injection technique with gadofosveset. Examples of intravascular T1 time with injection protocols in normal volunteers for the (**a**) 70 %/30 % bolus/infusion, (**b**) 60 %/40 %, (**c**) 100 % bolus, and a composite of all subjects imaged (**d**). Time zero indicates the beginning of infusion

As predicted by pharmacokinetic modeling (Fig. 3), the 100 % bolus (*n* = 3) had a lower T1 time initially but the change in T1 time was more than 10 % between arterial-venous equilibrium and 5 min thereafter. The average time of arterial-venous equilibrium was 79 s (71–94 min-max). Average T1 time over specific scan durations was not calculated because of the high degree of T1 deviation following equilibrium.

For the 70 %/30 % (*n* = 14) injection protocol designed to maintain up to 5 min steady state intravascular concentration, there was also a rapid dip in T1 time, followed by an average equilibrium time of 83 s (63–93 min-max). It was observed that the steady-state was maintained to sufficiently include a 5-minute scan duration following equilibrium with an average of 5.2 % (0.26–9.7 % min-max) variation in blood T1 time up to 7.5 min post equilibrium. Average T1 time during the 5-minute post-equilibrium scan duration was 269.9 ms ± 13.3, which differed from the predicted average T1 time of approximately 189.1 ± 1.5 (*P* <0.001).

For the 60 %/40 % protocol (*n* = 5) designed for a 5–10 min duration acquisition time, average equilibrium was reached at 85 s (66–95 min-max) and average change in T1 was under 10 % (2.3 % mean, 0.5–6.7 % min-max) up to 11.5 min following equilibrium. Average T1 time during the 10-minute post-equilibrium scan duration was 289.0 ms ± 9.0, which was higher than the predicted average T1 time of approximately 225.3 ms ± 4.8 (*P* <0.001).

In aggregate, 90 s was considered an acceptable estimate of the earliest appropriate time point of equilibrium for 100 %, 70 %/30 %, or 60 %/40 % injection protocols. Mean aortic pre-contrast T1 time was 1673 msec (1604–1743 95 % confidence interval) for all scanning instances, similar to the reported value of 1664 in the literature [15]. It is noted that the aggregate curves (Fig. 3d) for the sample are expected to appear more variable than intra-subject steady-state concentrations (Fig. 3a-c).

## Discussion

Using T1 mapping, the goal of this study was to evaluate the in vivo intravascular concentration of gadofosveset using bolus and dual injection techniques for variable time durations. There is recent interest in using gadofosveset for angiography at 3T [12–14, 18] which could improve contrast and signal in the coronary arteries compared to traditional GBCAs, which show comparably rapid loss of vascular signal at even limited scan durations (5–7 min). As predicted by mathematical modeling, the use of a dual injection technique with gadofosveset maintains a more stable intravascular concentration compared to the admittedly more simple single bolus technique. However, with the recent advances in coronary MRA using model-based reconstruction with 100 % imaging efficiency, scan duration can potentially be reduced to within 3–7 min [4–6]. These methods rely on a posteriori motion compensation techniques which benefit from high signal and contrast for non-rigid image-based registration [6]. With these goals, we used simulated pharmacokinetic data to predict the steady-state concentration-vs.time profile of MR contrast in humans. Specifically, we show the in vivo intravascular behavior of a dual injection technique for gadofosveset. Its most immediate application may be for coronary MRA [19]; however these methods are not exclusive to the anatomy studied, and may apply to other organs of interest. With proof-of-concept for the injection scheme, we offer an inclusive template for future steady-state intravascular concentrations for immediate or unanticipated applicability for contrast enhanced MRI. These findings may expand upon work focused on the newer contrast agent gadofosveset for cardiovascular applications such as myocardial T1 relaxation times and myocardial extracellular volume fraction calculations [20, 21].

Our experience with this injection scheme led us to make certain suggestions for accurate injection technique when using a dual-injector that uses the same line for saline and contrast (described in the Results section). Specifically, our technique is designed to achieve a number of goals: (1) Maximize subject comfort and safety; (2) Maximize injection accuracy; (3) Minimize contrast waste; (4) Minimize complexity; (5) Minimize setup time; (6) Maximize applicability to other injector types across institutions.

To the extent that the temporal resolution of an 11 heartbeat, breath-hold MOLLI sequence (1 scan every 40–60s) can determine, the earliest time point after bolus to begin scanning is suggested here. At approximately 90 s, steady state intravascular contrast concentrations should be sufficient for gadofosveset using dual-injection techniques.

The study was only focused on the intravascular concentration of contrast rather than addressing changes in the extravascular state. Once stable intravascular contrast concentrations are met, it may be possible to attain a more rigorous understanding of variations in extracellular flux of gadofosveset over the time of infusion and thereafter.

Regarding limitations of our study, we used 3T field strength and all scans used only healthy subjects. Our in vivo measurements demonstrated significantly higher average T1 time than the predicted pharmacokinetic model for scan durations of interest, which is likely in part due to the model’s inability to account for the time required for injection and arterial-venous equilibrium. This work was limited to the technical application of a dual-injection procedure and was not designed to systematically measure differences in diagnostic image quality. Although prediction of the absolute value of T1 time was not part of the research goal, the average pre-contrast T1 times in blood for our sample was comparable to that reported in the literature (1673 vs 1664, respectively). We cannot exclude a small amount of systematic bias, which may explain some of the differences in predicted T1 times compared to those measured in vivo. If lower T1 times approaching values below 200 ms for coronary artery MRA are desired, higher doses of gadofosveset may be required, which may warrant further investigation. The sample sizes for the 60 %/40 % and 100 % injection protocols were not as extensive as the 70 %30 % injection scheme in order to maximize evaluation of the more promising injection protocol (70 %/30 %), as predicted by pharmacokinetic modeling. Likely sufficient for proof-of-concept, this low sample size limited more broad sub-sample experimentation. Retrospectively, we observe that the injection of the entire contrast dose as a bolus may result in a comparably low intravascular concentration (Fig. 3d, 100 % curve) within 2–3 min after equilibrium compared to the other injection schemes. Although, we point out that it does so at the cost of the stability of concentration, we acknowledge that the bolus technique may be sufficient for image acquisition that does not require concentration stability. In these regards, further study would likely be required to compare objective measures of image quality in healthy and disease states.

## Conclusions

Pharmacokinetic simulation of gadofosveset intravascular concentration was exploited to formulate a dosing approach that optimized the imaging capabilities of this agent. We described a dual-injection technique, in which an initial gadofosveset bolus was followed by a slow infusion. This technique allowed for the rapid achievement of steady state intravascular gadofosveset concentrations, which remained stable for multiple scanning durations (5 and 10 min). Using this model, we provide proof-of-concept evidence in support of our injection technique in humans.

## Abbreviations

3T: 3.0 Tesla
DAo: descending aorta
ECG: electrocardiogram
GBCA: gadolinium-based contrast agent
GFR: glomerular filtration rate
LV: left ventricle
MOLLI: modified Look-Locker inversion recovery
MR: magnetic resonance
MRA: magnetic resonance angiography
MRI: magnetic resonance imaging
RA: right atrium
RV: right ventricle
